# Synergistic enhancement of hydrophobic dental adhesives: autonomous strengthening, polymerization kinetics, and hydrolytic resistance

**DOI:** 10.3389/fdmed.2024.1373853

**Published:** 2024-04-26

**Authors:** Burak Korkmaz, Erhan Demirel, Qiang Ye, Anil Misra, Candan Tamerler, Paulette Spencer

**Affiliations:** ^1^ Institute for Bioengineering Research, University of Kansas, Lawrence, KS, United States; ^2^ Canon Virginia, Inc., Newport News, VA, United States; ^3^ Department of Civil and Environmental Engineering, Florida International University, Miami, FL, United States; ^4^ Department of Mechanical Engineering, University of Kansas, Lawrence, KS, United States; ^5^ Bioengineering Program, University of Kansas, Lawrence, KS, United States

**Keywords:** dental adhesive, hydrolytic degradation, highly crosslinked network, sol-gel reaction, dynamic mechanical analysis, hydrophobic resins

## Abstract

The leading cause of composite restoration failure is recurrent marginal decay. The margin between the composite and tooth is initially sealed by a low-viscosity adhesive, but chemical, physical, and mechanical stresses work synergistically and simultaneously to degrade the adhesive, destroying the interfacial seal and providing an ideal environment for bacteria to proliferate. Our group has been developing self-strengthening adhesives with improved chemical and mechanical characteristics. This paper reports a self-strengthening adhesive formulation that resists hydrolysis-mediated degradation by providing intrinsic reinforcement of the polymer network through synergistic stimulation of free-radical polymerization, sol-gel reaction, and hydrophobicity. Hydrophobic resin formulation (NE1) was developed using HEMA/BisGMA 28/55w/w and 15 wt% MPS. Control (NC1) contained HEMA/BisGMA 28/55 w/w and 15 wt% MES. The polymerization kinetics, water sorption, leachates, and dynamic mechanical properties of the resin samples were investigated. The NC1 and NE1 samples showed comparable polymerization kinetics, degrees of conversion and water sorption. In contrast, NC1 showed significantly higher levels of HEMA and BisGMA leachate, indicating faster degradation in ethanol. At day 3, cumulative HEMA leachate for NC1 was ten times greater than NE1 (*p < 0.05*). Dynamic mechanical properties were measured at 37 and 70°C in both dry and wet conditions. Under dry conditions, the storage moduli of NC1 and NE1 were comparable and the glass transition temperature (*T*_g_) of NC1 was statistically significantly lower (*p* < 0.001) than NE1. Under wet conditions, the storage modulus of NC1 was lower than NE1 and at 70°C there was a threefold difference in storage modulus. At this temperature and under wet conditions, the storage modulus of NC1 is statistically significantly lower (*p* < 0.001) than NE1. The results indicated that in the wet environment, NE1 provided lower chain mobility, higher crosslink density, and more hydrogen bonds. The newly formulated methacrylate-based adhesive capitalizes on free-radical polymerization, sol-gel reactions, and hydrophobicity to provide enhanced mechanical properties at elevated temperatures in wet environments and hydrolytic stability under aggressive aging conditions.

## 1 Introduction

Despite the growing popularity and nearly sixty years of research, composite restorations fail faster than amalgam under a variety of circumstances (1–3). Composite materials are technique sensitive—composite restorations will likely fail when isolation is challenging and the operatory field is contaminated (4). Composite restorations fail primarily due to secondary caries and fracture (5) — patient factors such as caries risk and parafunctional habits are a major factor in composite restoration failure (6). Indeed, the potential for composite restoration failure is two to three-times greater for patients with high and medium caries risk/susceptibility (7). Factors impacting patient's caries risk/susceptibility include socioeconomic factors, access to care, biological and behavioral factors (8). For example, high-risk patients include the 4 million U.S. children (9) and more than 100 million adults (10) who do not receive regular dental care. Physical and biological risk factors include insufficient salivary flow, saliva composition, inadequate fluoride exposure, increased concentrations of cariogenic bacteria, and gingival recession (8). Behavioral risk factors include poor oral hygiene, inappropriate dietary habits, frequent and persistent consumption of oral medications containing sugar (8).

The increased susceptibility of composite restorations to secondary decay is twice as great at the gingival margin of Class II and V restorations (11). Recurrent decay at these margins is related to increased plaque accumulation, biofilm stagnation, and inadequate adaptation of the restorative material (11). The low-viscosity adhesive that bonds the composite to the tooth is intended to seal the composite/tooth interface and provide a durable barrier to noxious agents. However, the fragile adhesive seal to dentin is readily damaged by acids, enzymes, and oral fluids. Bacteria and bacteria by-products infiltrate the resultant marginal gaps, destroying the tooth structure and accelerating erosion of the adhesive (12).

Water is ubiquitous in the mouth and a constant threat to the durability of resin-based materials. Fortunately, nature offers inspiration for achieving adhesives with strong cohesive strength in caustic, wet environments (13). Leveraging lessons from nature, our research group has developed methacrylate-based adhesives that capitalize on free-radical polymerization (FRP) and sol-gel reaction to provide adhesives with autonomic strengthening properties (14–16). The composition of these novel dental adhesives included HEMA, BisGMA, and γ-methacryloxyproyltrimethoxysilane (MPS) as well as the 3-component photoinitiator system. In brief, when the liquid resin was irradiated by visible light, the polymethacrylate network was produced by free-radical polymerization of BisGMA and HEMA. Simultaneously, the alkoxysilane groups were hydrolyzed in a reaction catalyzed by the photoacid produced during the visible-light irradiation. The processes led to polysiloxane chains interconnected in the polymethacrylate matrix. The autonomous hydrolysis and condensation of the alkoxysilyl moieties continued when the resin was soaked in water or lactic acid. The resulting silanol groups reacted with the hydroxyl groups of HEMA or BisGMA to form covalent bonds (14). The results showed that the MPS molecule with its trimethoxy silane and methacrylate functionalities contributed to the enhanced stability and mechanical properties of the adhesive formulation (14).

While we have reported the beneficial effects of the self-strengthening approach, i.e., enhanced mechanical properties and degradation resistance when aged in water or lactic acid, the formulations were not representative of hydrophobic resins. For example, we examined the effect of composition, i.e., percent silane monomer, initiator system, and solvent (water), on the sol-gel reaction and the concomitant properties of the adhesive (14). As shown in Table 1, the hybrid polymer that coupled visible-light induced sol-gel reaction with free radical polymerization showed enhanced hydrolytic stability, mechanical, and thermal properties as compared to a model methacrylate-based adhesive. In 2020, we studied the time-dependent mechanical properties of methacrylate-based model adhesives with and without γ-methacryloxypropyl trimethoxysilane (MPS). The mechanical behavior and network structure were significantly dependent on the autonomous strengthening reaction under wet conditions. The results showed higher degree of conversion, lower leachate, and enhanced resistance to deformation in the MPS-containing model adhesive (15). In 2022, we examined the effect of a relatively hydrophilic formulation with a low crosslink ratio on the sol-gel reaction and mechanical properties (16). We postulated that the hydrophilic properties in combination with relatively low crosslink density could facilitate free radical polymerization and the sol–gel reaction. The mechanical properties of the MPS-containing formulation showed further improvement following aqueous aging (16).

**Table 1:** Comparison of properties of former formulations.

Sample code	DC%	*W*_sp_%	Storage modulus (MPa)	HEMA leachate (μg/ml)	BisGMA leachate (μg/ml)	References
E1-3PI^a^	69.5 (0.2)	10.53 (0.03)^d^	359.4 (4)^e^	589 (25)^g^	240 (3)^g^	(14)
E2-3PI^a^	72.0 (0.1)	9.80 (0.08)^d^	557.1 (23.4)^e^	307 (21)^g^	124 (8)^g^	(14)
E1^b^	61.8 (0.4)	15.5 (0.1)	–^f^	44 (4)^h^	–	(15)
E1^c^	88.3 (1.4)	18.6 (1.6)	13.3 (0.4)^e^	1,473 (12)^i^	–	(16)

^a^ E1-3PI: [HEMA/BisGMA − 45/55(w/w)] − 95% + MPS − 5% (w). E2-3PI: [HEMA/BisGMA − 45/55(w/w)] − 90% + MPS − 10% (w) with 4% of PI system were used with respect to total mass of monomers.

^b^ E1: HEMA − 58% + BisGMA − 30% + MPS − 10% + 3PI − 2%.

^c^ E1: HEMA − 73% + BisGMA − 15% + MPS − 10% + 3PI − 2%.

^d^ Water miscibility.

^e^ Storage Modulus at 70°C in wet conditions.

^f^ No wet DMA analysis in this study.

^g^ Cumulative HEMA Leachate plateaued at 7th day, BisGMA leached on the 56th day in ethanol.

^h^ 24 h water incubation at 37°C.

^i^ 4 days water incubation at 37°C.

The control formulations in the 2020, 2022, and current investigation contained HEMA, BisGMA, and methacryloxyethoxy trimethyl silane (MES) as well as the 3-component photoinitiator system. While the control experienced free radical polymerization of HEMA and BisGMA leading to the polymethacrylate network, the trimethylsilane group in the MES lacks the ability to undergo the hydrolysis-polycondensation reactions (16). The MES-containing formulation (NC1) does not generate hydroxyl groups—MES does not contribute to the crosslinking and network evolution via hydrogen bonding (15).

Rad and colleagues reported that controlling water sorption and protecting against hydrolytic degradation is at the forefront of research in methacrylate-based dental adhesives (17). Recent literature has reported significant benefits, including increased bond strength and reduced nanoleakage, when vulnerable bonding interfaces are protected by hydrophobic resin coatings (18). Based on these recent observations, hydrophobic resins could lead to dental adhesives that provide a durable barrier at the composite/tooth interface.

To our knowledge, the current study marks the first investigation of the impact of the sol-gel reaction on the structure and property relationships in a hydrophobic model methacrylate-based adhesive. The alkoxysilane groups will likely be hydrolyzed in a reaction catalyzed by the photoacid produced during the visible-light irradiation. It is, however, postulated that the hydrophobic characteristics, low water sorption, and high crosslinker concentration will inhibit mobility of methoxysilyl functional groups and concomitantly, the autonomous strengthening reaction. The aims of the current investigation were to study the polymerization behavior, degradation resistance, and dynamic mechanical properties of hydrophobic methacrylate-based adhesives with or without γ-methacryloxypropyl trimethoxy silane (MPS). Resistance to degradation was studied following ethanol aging and dynamic mechanical properties of dry and water-saturated samples were studied at 37 and 70°C.

## 2 Materials and methods

### 2.1 Materials

**Figure 1:**
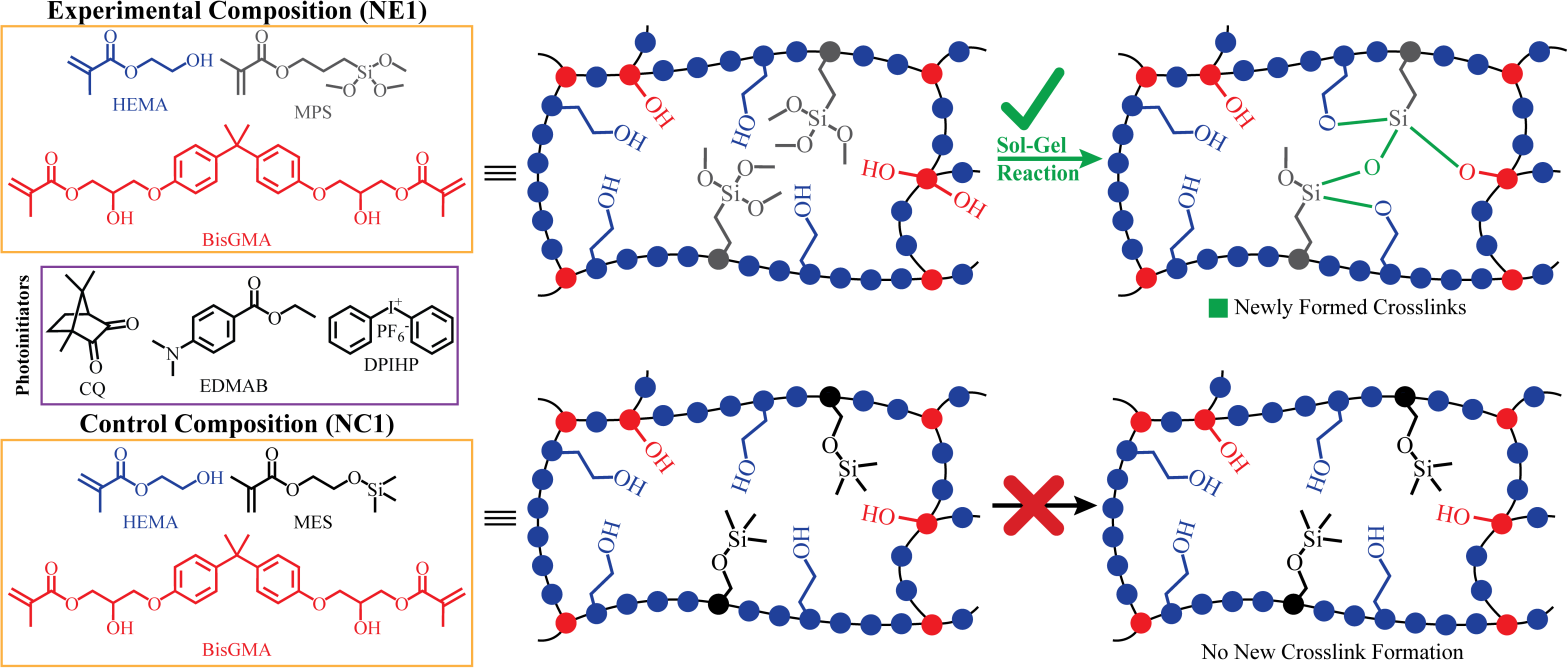
Chemical structures of components in formulations and illustration of the envisioned network structure and the inherent self-strengthening mechanism.

The following monomers and photoinitiators were purchased from Sigma-Aldrich (St. Louis, MO, USA): 2-hydroxyethyl methacrylate (HEMA), bisphenol A glycerolate dimethacrylate (BisGMA), diphenyliodonium hexafluorophosphate (DPIHP), camphoroquinone (CQ), ethyl-4-(dimethylamino) benzoate (EDMAB), methacryloxyethoxy trimethyl silane (MES), and γ-methacryloxypropyl trimethoxy silane (MPS). All materials were used as received without further purification. The chemical structures of the monomers and photoiniators are presented in Figure 1.

### 2.2 Preparation of adhesive formulations

A 3-component photoinitiator system was used (CQ-EDMAB-DPHIP (0.5/0.5/1 wt/wt/wt) for each resin formulation (19). All the mixtures were prepared under amber light in brown glass vials (14). This procedure was necessary to avoid premature polymerization. For each formulation HEMA and the organosilanes MES (for NC1) and MPS (for NE1) were added to amber vials, the photoinitiators were added and the solutions were mixed thoroughly to obtain homogeneous mixtures. BisGMA was added to the mixture and the formulations were stirred and shaken for 24 h at room temperature (23 ± 2°C). The HEMA/BisGMA/MES (28/55/15) formulation was used as the control (NC1) and HEMA/BisGMA/MPS (28/55/15) formulation as the hydrophobic resin formulation (NE1). The formulations are listed in Table 2.

**Table 2:** Chemical composition of the formulations and their values of degree of conversion (DC), maximum polymerization rate (*R*_p_^max^/[*M*]), surface roughness (*R*_a_), contact angle (CA) and water sorption^a^.

Run	H/B (wt%)^b^	MES (wt%)	MPS (wt%)	3PI (wt%)	*W*_sp_ (wt%)	CA (degrees°)	DC (%)	*R*_p_^max^ (1/s)	*R*_a_ (μm) Incubation in ethanol		
									Before incubation	Day 9	Day 16
NC1	83	15	0	2	7.82^c^ (0.65)	57.62^c^ (1.44)	65.4^c^ (0.5)	11.0^c^ (2.5)	3.474^c^ (0.280)	1.292^c^ (0.207)	1.227^c^ (0.254)
NE1	83	0	15	2	7.28^c^ (0.51)	62.18^d^ (2.33)	65.7^c^ (0.8)	11.1^c^ (2.8)	2.930^c^ (0.713)	0.758^d^ (0.056)	0.800^d^ (0.024)

^a^ The values in parentheses indicate the standard deviation.

^b^ The weight percentage of HEMA/BisGMA:28/55, Photoinitiators-CQ-EDMAD-DPIHP:0.5–0.5–1.

^c,d^ The different letters after mean values indicate significant differences, same letter indicates no significant difference between values of NC1 and NE1 (*p* < 0.05).

### 2.3 Real-time double bond conversion and maximum polymerization rate

#### 2.3.1 Fourier transform infrared spectroscopy (FTIR)

FTIR was used to determine the degree of conversion (DC) (20) and polymerization rate. The infrared spectrometer (Frontier FTIR Spectrometer, Perkin-Elmer, Waltham, MA) was used at 4 cm^−1^ of spectral resolution and wavenumber range of 650–4,000 cm^−1^ to continuously monitor the photopolymerization *in situ*. The infrared spectrometer is equipped with software (Spectrum TimeBase v3.0, Perkin-Elmer, Waltham, MA) that allows continual scans to be taken with 4 s intervals. Therefore, the DC as a function of time can be determined. The conversion of methacrylic C = C double bond was monitored by using 1,637 cm^−1^ (C = C)/1,714 cm^−1^ (carbonyl) as the band ratio profile (21). The DC values were calculated according to the equation, DC = (1-*R*_p_/*R*_R_) × 100, where *R*_p_ and *R*_R_ are the band ratios for adhesive after polymerization and before polymerization, respectively. The reported value of DC is the average of the last 30 values of the time-based spectra when the DC values reach a plateau.

Approximately 5–10 µl of each adhesive formulation was poured on the crystal of the attenuated total reflectance (ATR) accessory (Universal ATR Sampling Accessory, Perkin-Elmer, Waltham, MA). The adhesive was covered by Mylar film to avoid interference from ambient oxygen and moisture. The adhesive was exposed to visible curing light (Spectrum 800, Dentsply, Milford, DE) with 550 mW/cm^−2^ of intensity after the first 120 s of time-based analysis. The adhesive was exposed to visible light for 40 s and the IR spectra were recorded in 4 s intervals for ∼3 h. Three measurements were recorded for each formulation.

The kinetic data of polymerization was obtained by calculating the first derivative of degree of conversion against time (*R*_p_^max^). DC and *R*_p_^max^ values are listed in Table 2 (22, 23).

### 2.4 Water sorption

Round disc samples (1.2 mm × 4 mm diameter) were prepared for the leachate and water sorption studies. Homogeneous mixtures of the adhesives were added to cylindrical 1 ml syringes (BD, Becton, Dickinson and Company, Franklin Lakes, NJ, USA). Great effort was taken to avoid air bubbles during the introduction of the formulations into the syringes. The filled and sealed syringes were placed in the LED Curebox (LED Curebox, 100 mW/cm^2^ irradiance, Proto-tech, Portland, OR) to undergo polymerization via visible-light exposure for 40 s. Following light-polymerization, the syringes were stored in the dark for a minimum of 48 h. After light polymerization and dark cure, disc samples were prepared by sectioning the syringes to the intended thickness using a Buehler Isomet 1000 Precision Saw. The resulting disc samples were prewashed by submerging them in 2 ml of water for seven days. Following the prewash, the samples were thoroughly dried under vacuum until a constant mass was achieved, m_1_. After the mass plateaued, five disc samples for each formulation were submerged in 2 ml of ultrapure water and weighed at time intervals 0, 1, 2, 4, 6, 8, 12, 24, 36, 48, 72, 96, and 120 h or until they reached constant mass, m_2_. Water sorption was calculated according to the equation below.


$$W_{\mathrm{sp}}\text{\%}=\frac{m_{2}-m_{1}}{m_{1}}\times 100$$

### 2.5 Contact angle study

High-grade V1 mica discs (TED PELLA, Inc.) were utilized as substrates for resin application. A volume of 50  µl of resin was dispensed onto each mica disc. The discs were then subjected to a spin coating process using a Laurell Model WS-400BZ-6NPP/LITE Spin Coater (Laurell Technologies Corporation, Lansdale, PA, USA) at 2,000 rpm for 30 s under a nitrogen atmosphere to ensure uniform coating. Post spin coating, the resin-coated mica discs were transferred to an LED Curebox (100 mW/cm^2^ irradiance, Proto-tech, Portland, OR) for polymerization. The photopolymerization was conducted through visible-light exposure for a duration of 40 s. The coated discs were stored in a dark environment for a minimum of 48 h prior to further analysis. The surface contact angle properties of the resin-coated mica discs were assessed using an Attension Theta Optical Tensiometer (Biolin Scientific, Stockholm, Sweden). 10 μl of Milli-Q ultrapure water was employed as the testing liquid to measure the contact angles on the NE1 and NC1 coated mica discs. Four measurements have been conducted for each formulation.

### 2.6 Dynamic mechanical analysis (DMA)

The viscoelastic properties of the two resin formulations were characterized using a DMA Q800 (TA Instruments, New Castle, USA) equipped with a cooling accessory operated with liquid nitrogen. A standard 3-point bending clamp was used for the vacuum-dried beam samples and a 3-point bending submersion clamp was used for the water-submerged beam samples. Five rectangular beam samples (1 mm × 1 mm × 15 mm) per group were prepared for each formulation by injecting 30–40 μl of the liquid resin formulations at room temperature into glass tubing (Vitrocom Technical Glass, borosilicate, 8100 Square VitroTubes™) and light-curing for 40 s using an LED light-curing box (LED curedome, 100 mW/cm^2^ irradiance, Prototech, Portland, OR). After a 1 h dark cure, beam samples were removed from the glass molds and aged in water to promote the hydrolysis reaction at 37°C for 7 days. This step was followed by incubation at 37°C for 48–72 h to promote the condensation reaction (22). Ten beam samples for each formulation were randomly divided into two groups for testing under dry and wet conditions, respectively. To dry the beam samples, they were placed under vacuum at 37°C for at least 96 h or until they reached constant mass. The DMA test was conducted in the temperature range 20–180°C with a ramping rate of 3°C/min at a frequency of 1 Hz. The water-submerged beam samples were incubated in ultrapure water at 37°C until they reached a constant mass. Samples were placed on 3-point bending submersion clamp and tested in a temperature range of 10–70°C with 1.5°C/min at a frequency of 1 Hz. The support span length for the DMA tests was 10 mm (15).

### 2.7 Thermal gravimetric analysis

Thermal degradation properties of the resins were examined by heating the resin samples weighing ∼3 mg for both formulations from 25°C to 600°C using Pyris 1 TGA Thermogravimetric Analyzer (PerkinElmer, Waltham, MA, USA) with a temperature ramp of 10°C per minute under nitrogen atmosphere.

### 2.8 Leachable study: degradation in ethanol

Five disc samples for each formulation were prepared using the approach described under Section 2.4 (Water Sorption). After the dried disc samples reached constant mass, they were submerged in 1 ml ethanol (HPLC Grade). The storage solutions were collected every 24 h for the first seven days and every 72 h after day 7. Fresh ethanol was added after every collection. The concentration of leachate in the storage solutions was analyzed using high performance liquid chromatography (HPLC) on a system (Shimadzu LC-2010C HT, software EZstart, version 7.4 SP2) equipped with 250 × 4.6 mm column packed with 5 μm C-18 silica (Luna, Phenomenex Inc., Torrance, CA). The mobile phase was acetonitrile/water with 0.1 trifluoroacetic acid (TFA) (Gradient flow from 15/85 v/v to 100/0 v/v in 56 min). The system was operated as follows: 1 ml/min flow rate, detection at 208 nm, 20 μl sampling volume, and 40°C. The column was calibrated using known concentrations of BisGMA, HEMA, MPS, and EDMAB. The calibration curves of BisGMA (Linear Fitting of BisGMA (2.5–250 μg/ml, *R*^2^ = 0.9995), HEMA (Linear Fitting of HEMA (2.5–250 μg/ml, *R*^2 ^= 0.9998), MPS (Linear Fitting of MPS (2.5–125 μg/ml, *R*^2^ = 0.9997), and EDMAB (Linear Fitting of EDMAB (2.5–100 μg/ml, *R*^2^ = 0.9999) were used to calculate the concentration of these species in the storage solutions. The concentration calculation was based on the intensity of the chromatographic peaks at the corresponding retention time (minutes) for HEMA, MPS, EDMAB and BisGMA, which are 9.3, 10.2, 29.0, and 38.5 min, respectively.

Surface roughness (*R*_a_) scans were conducted using a Wyko NT1100 noncontact optical profilometer (Veeco Instruments) at 10× magnification. Instrument was calibrated with Step Height Standard of Veeco (Calibrated Step Height Value: 8.353 um) before performing scans. For each formulation, two of the five disks used in the Ethanol Leachable Study were randomly selected for scanning. Five different regions of the surface were scanned at baseline (before ethanol aging) and at days 9 and 16 following aging in ethanol.

### 2.9 Statistical analysis

The results from the following experiments: water sorption, degree of conversion (FTIR), rate of polymerization, and accumulative concentration of leachates (HPLC) were analyzed using one-way analysis of variance (ANOVA) together with Tukey's test at *α* = 0.05 (Microsoft Excel Microsoft 365, Microsoft Corporation. Redmond, Washington, USA). Statistical analysis of dynamic mechanical analysis (DMA) was conducted with an unpaired parametric *t*-test with Welch's correction using GraphPad Prism (version 10.1.0 for Windows, GraphPad Software, Boston, Massachusetts, USA), and APA style *p-values* were reported. Statistical analyses were used to identify significant differences in the means.

## 3 Results

**Figure 2:**
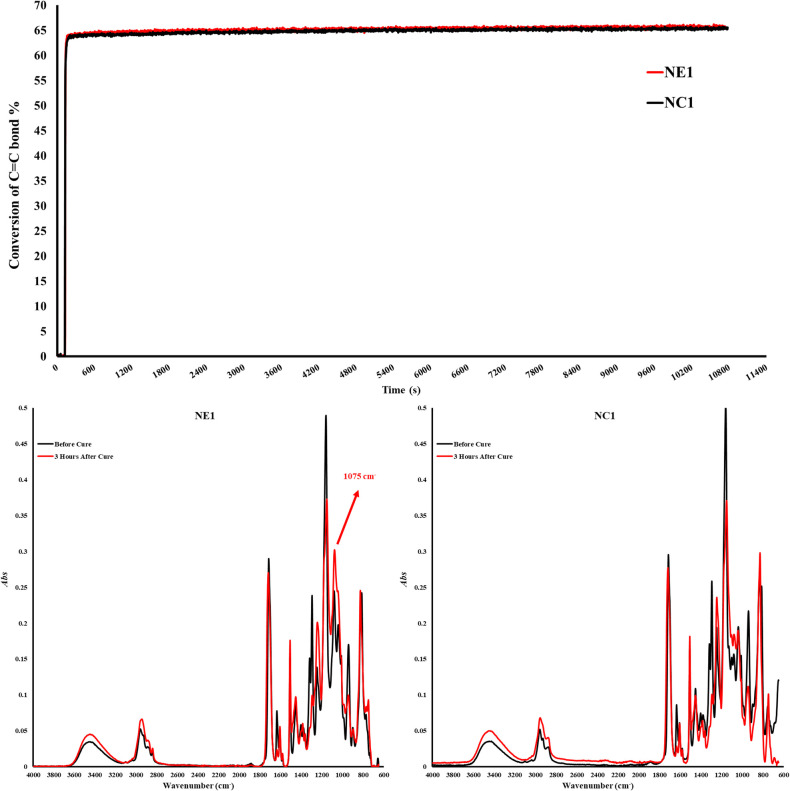
Degree of conversion graph and FTIR Spectra of NC1 and NE1 before and after photopolymerization.

The degree of conversion and maximum polymerization rate (*R*_p_^max^) are shown in Table 2. The degree of conversion (DC) of the control (NC1) and experimental (NE1) formulations are comparable (*p > 0.*05) at 65.4 ± 0.5 and 65.7 ± 0.8%, respectively (Figure 2). There is no significant difference (*p > 0.*05) between the maximum polymerization rates (*R*_p_^max^) of the NC1 and NE1 formulations at 11.0 ± 2.5 and 11.1 ± 2.8, respectively.

**Figure 3:**
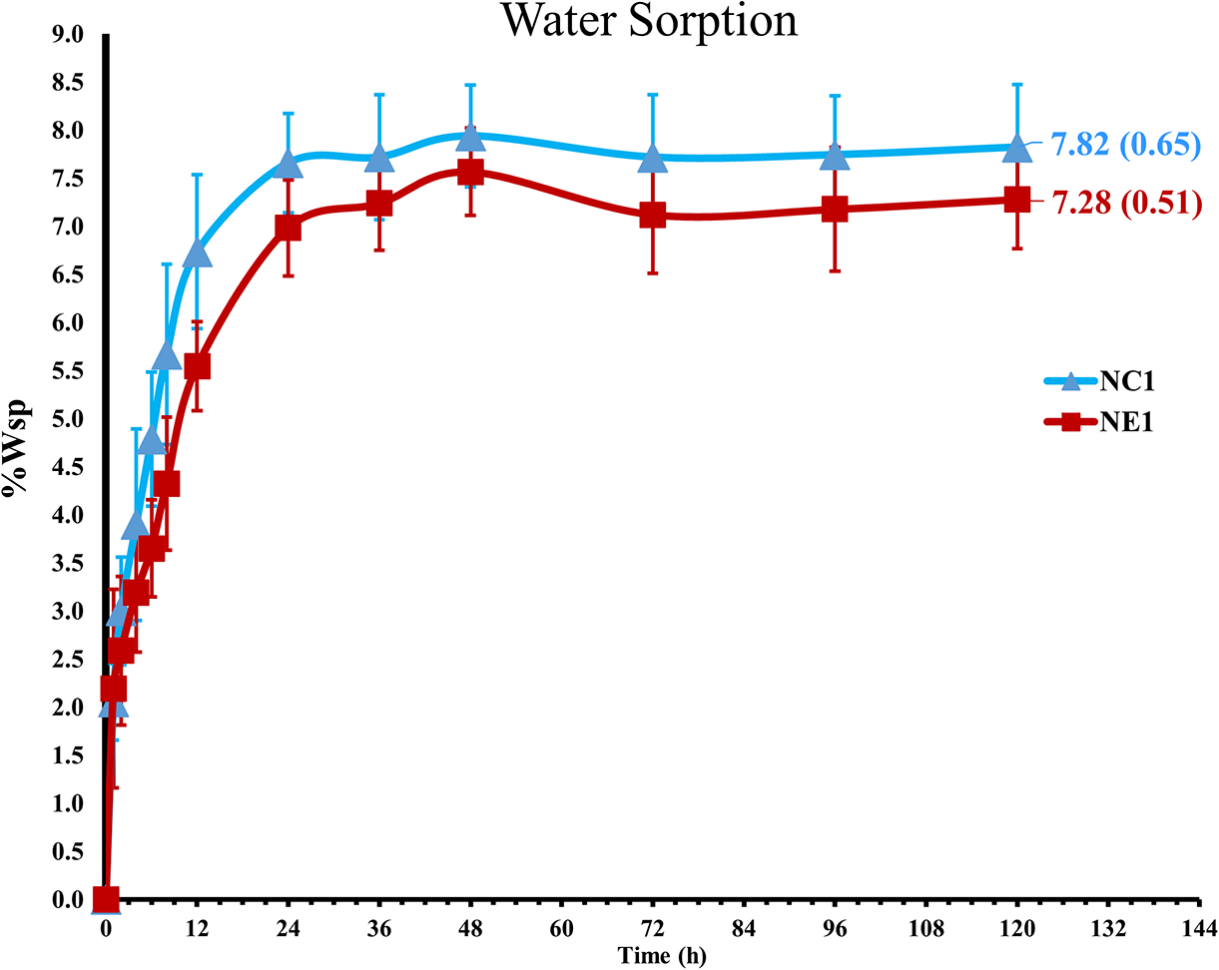
Water sorption of the NC1 and NE1.

The ability of the solid copolymers to retain water was investigated and Figure 3 demonstrates the water sorption kinetics for the NC1 and NE1 formulations. Water sorption increased gradually and plateaued after 36–48 h storage at 37°C. As shown in Table 2; Figure 3, NC1 showed slightly higher water sorption at 7.82 ± 0.65% as compared to NE1 (7.28 ± 0.51%) but the difference was not statistically significant (*p *> 0.05).

The contact angle values for the NC1-coated disc are lower than NE1-coated disc at 57.62 ± 1.44° and 62.18 ± 2.33°, respectively. The contact angle values for NC1 are statistically significantly lower (*p* < 0.05) than NE1 (Table 2).

**Figure 4:**
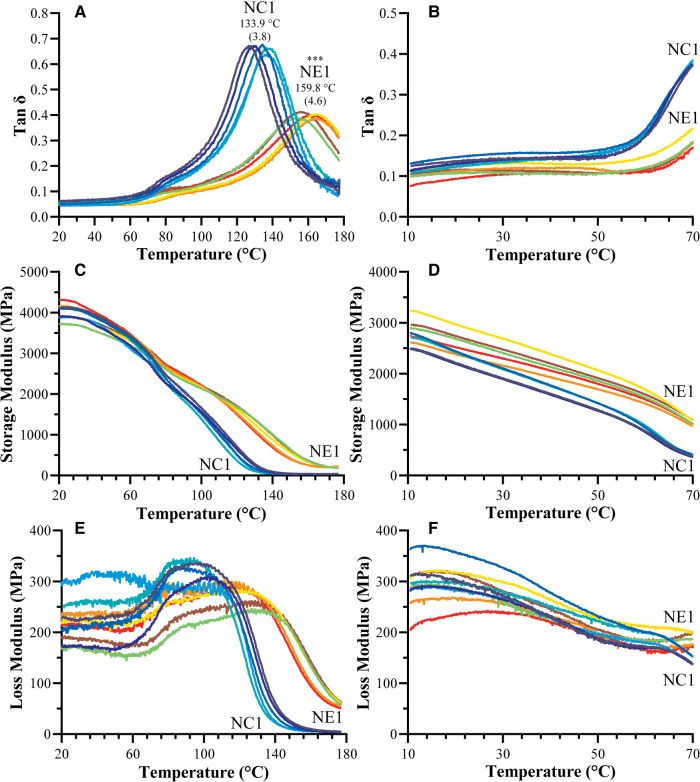
Representative tan δ vs. temperature curves for NC1 and NE1 (**A**) vacuum dried and (**B**) water-submersed samples, storage modulus vs. temperature curves for NC1 and NE1 (**C**) vacuum dried and (**D**) water-submersed samples, and loss modulus vs. temperature curves for NC1 and NE1 (**E**) vacuum dried and (**F**) water-submersed samples (***denotes *p* < .001 (*n* = 5, ±SD) for NE1 against NC1 for corresponding analysis.

The dynamic mechanical properties of the NC1 and NE1 formulations under dry and wet conditions are shown in Figure 4 and summarized in Table 3. The tan δ vs. temperature plots in Figure 4A revealed distinct glass transition temperature (*T*_g_) values for both formulations under dry conditions. NE1 exhibited a significantly higher (*p* < 0.001) *T*_g_ (159.8 ± 4.6°C) than NC1 (133.9 ± 3.8°C). The storage modulus plots in Figures 4C,D ascended to 180°C for dry testing, whereas it only reached 70°C for wet testing because of the temperature limit of the 3-point bending submersion clamp. The storage modulus, a measure of material stiffness, demonstrated a general decreasing trend with increasing temperature for NC1 and NE1 under both dry and wet conditions. Under dry conditions, the storage moduli of NC1 and NE1 are comparable (Table 3) with the exception of the rubbery region above 175°C. In this region, the storage modulus of NE1 was significantly greater (202.0 MPa) than NC1 (36.5 MPa) for the vacuum-dried samples. Under wet conditions, the storage moduli of NE1 are higher than NC1, and the difference is particularly marked at 70°C. At this temperature and under wet conditions, there is more than a threefold difference in storage moduli. The storage modulus of NE1 (1,007.2 ± 43.4 MPa) is significantly greater (*p* < 0.001) than NC1 (362.1 ± 29.2 MPa) at 70°C under wet conditions (Table 3).

**Table 3:** Values of the storage modulus (*E*′) of vacuum dried and water-submersed samples at various temperatures.

		Storage modulus (MPa)				Glass transition, *T*_g_, °C	tan(δ)
		25°C	37°C	70°C	Rubbery modulus >175°C^a^		
Vacuum dried	NC1	4,018.8 (116.7)	3,892.3 (104.0)	2,880.2 (140.0)	36.5 (1.5)	133.9 (3.8)	0.6621 (0.01572)
	NE1	3,989.2 (232.6)	3,850.1 (195.1)	3,049.0 (81.1)	202.0 (15.7)^b^	159.8 (4.6)	0.3982 (0.01079)
Water-submerged	NC1	2,113.9 (117.2)	1,737.1 (94.9)	362.1 (29.2)	–		–
	NE1	2,522.4 (209.2)^c^	2,210.7 (174.4)^d^	1,007.2 (43.4)^b^	–		–

^a^ Average of last 20 points recorded.

Superscript letters indicate significant differences between NE1 and NC1 values at relative temperatures (^b^*p* < .001, ^c^*p* = .008, ^d^*p* = .002).

**Figure 5:**
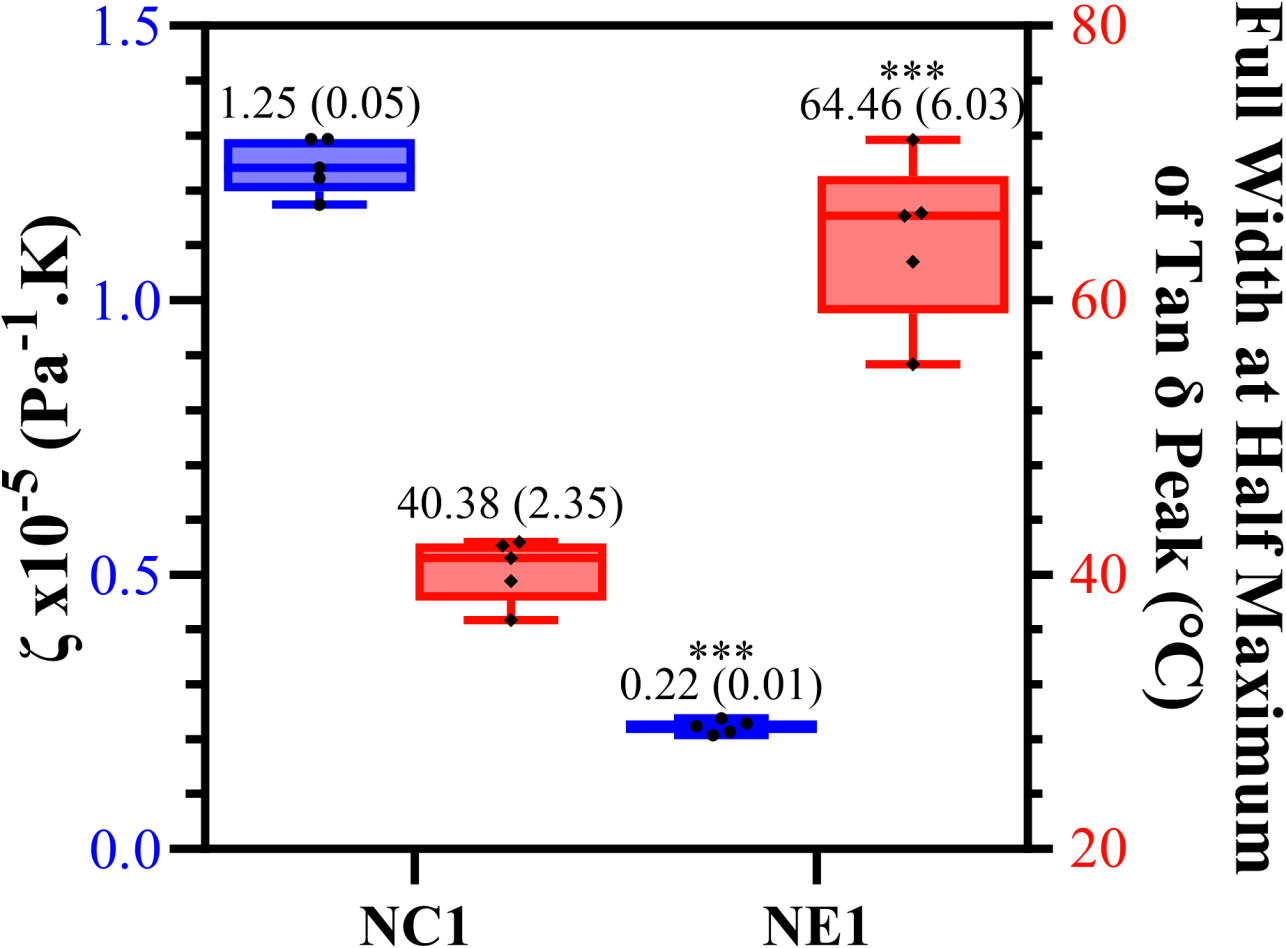
Box plot showing minimum to maximum values for NE1 and NC1 samples for the crosslinking density (blue) and full width at half maximum values of tan δ peak (°C) (red) for vacuum dried samples (***denotes *p* < .001 (*n* = 5, ±SD) for NE1 against NC1 for corresponding analysis.

The calculated values of relative crosslink density (ζ) and corresponding full-width-half-maximum (FWHM) values are shown in Figure 5. The significantly lower (*p* < 0.001) ζ value for NE1 (0.22 × 10^−5^ Pa^−1^K) compared to NC1 (1.25 × 10^−5^ Pa^−1^K) indicates increased crosslink density in the NE1 formulation. The FWHM value of NE1 (64.46 ± 6.03) was significantly greater than NC1 (40.38 ± 2.35) indicating a more heterogeneous polymer network.

**Figure 6:**
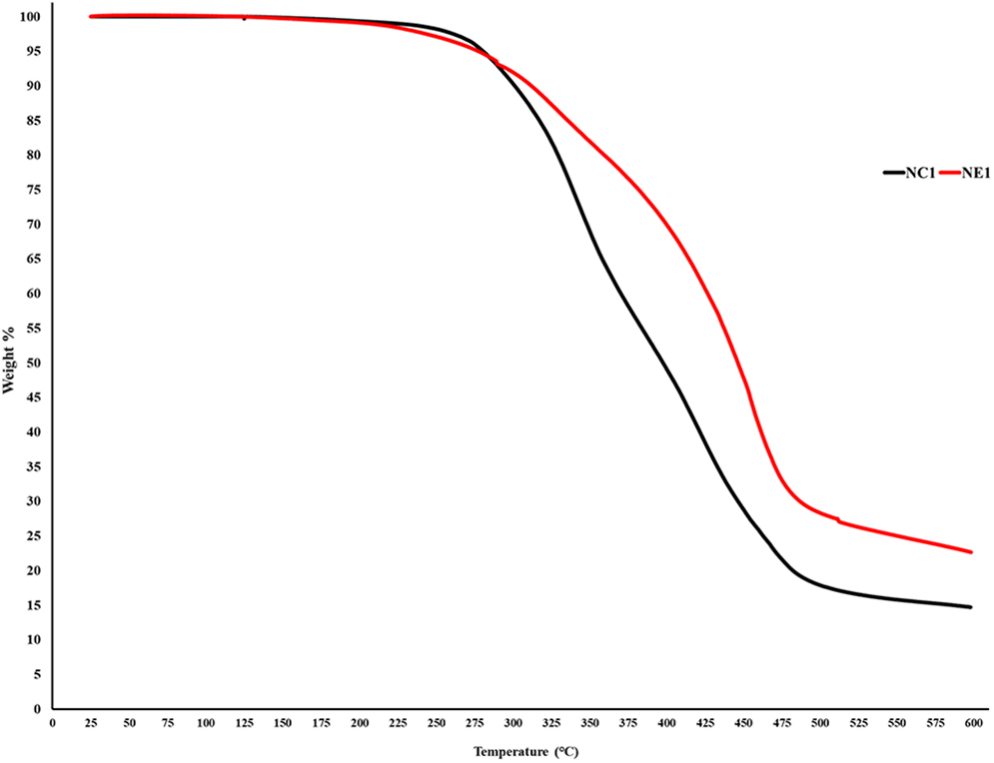
TGA thermograms of NC1 and NE1.

Thermal degradation profiles for NC1 and NE1 resin samples are shown in Figure 6. Approximately 10% of the NC1 resin degrades between 200 and 300°C. The major degradation of the NC1 resin occurs after 300°C with NC1 losing 77% of its weight at 500°C. In comparison, about 10% of the NE1 resin degrades between 220 and 300°C. NE1 loses 55% of its weight at 510°C.

**Figure 7:**
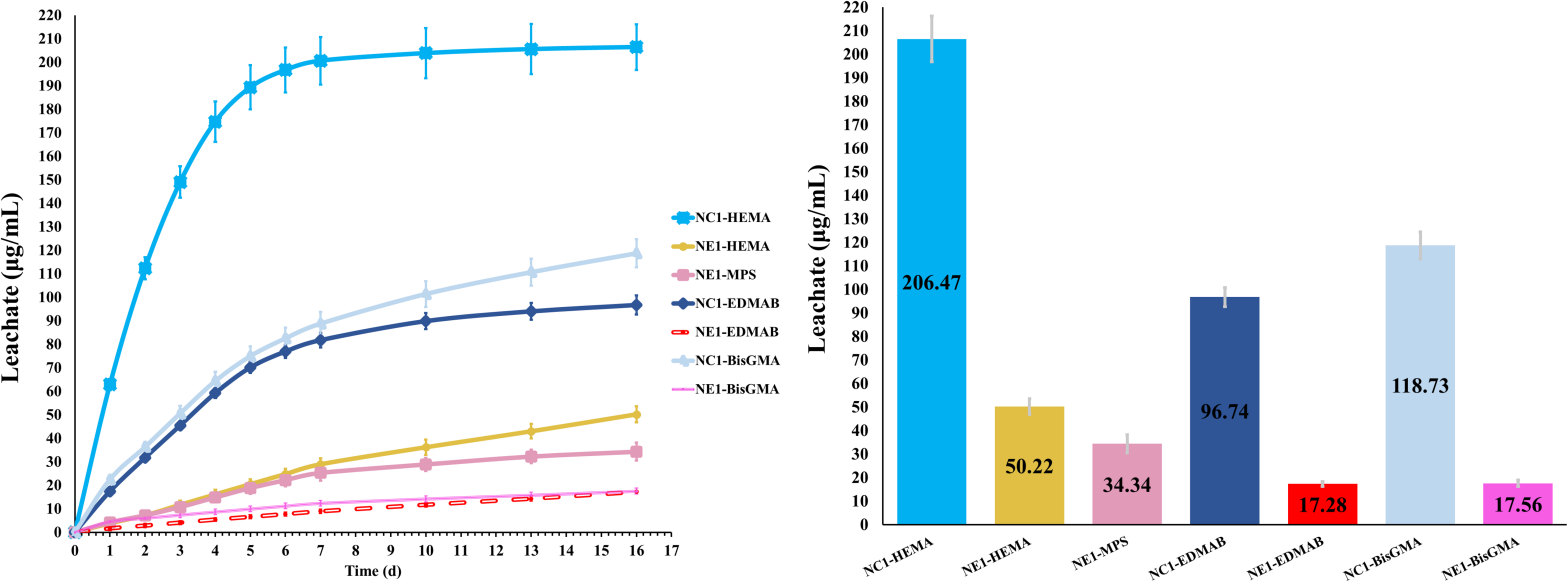
Average cumulative leachate concentrations of components from NC1 and NE1.

The average cumulative concentration of leachates from the samples stored in ethanol at room temperature (23 ± 2°C) is shown in Figure 7. The cumulative values for leached species, i.e., HEMA, MPS (NE1), EDMAB, and BisGMA, were calculated by comparing the peak intensities of the chromatographs of the storage solutions to the calibration curves of standard solutions. The average cumulative value for MPS leached from ethanol-stored NE1 samples was 34.34 μg/ml. The average cumulative value for MES could not be determined because of significant peak overlap with HEMA. The cumulative leachates for HEMA, EDMAB, and BisGMA are significantly different (*p < 0.05*) for NC1 and NE1. The average cumulative value for HEMA leached from ethanol-stored samples was 206.47 ± 9.75 and 50.42 ± 3.36 μg/ml for NC1 and NE1, respectively. The average cumulative values for EDMAB and BisGMA leached from ethanol-stored samples were considerably lower than HEMA. EDMAB leachates were 96.74 ± 4.06 and 17.28 ± 1.09 μg/ml for NC1 and NE1, respectively. BisGMA leachates were 118.73 ± 5.90 and 17.56 ± 1.33 μg/ml for NC1 and NE1, respectively. Figure 7 shows the kinetic behavior of the degradation of ethanol-stored NC1 and NE1 samples from 0 to 16 days. The amount of the leached species was reduced precipitously after the first week. The cumulative values for leached HEMA reached a plateau after 7–10 days.

**Figure 8:**
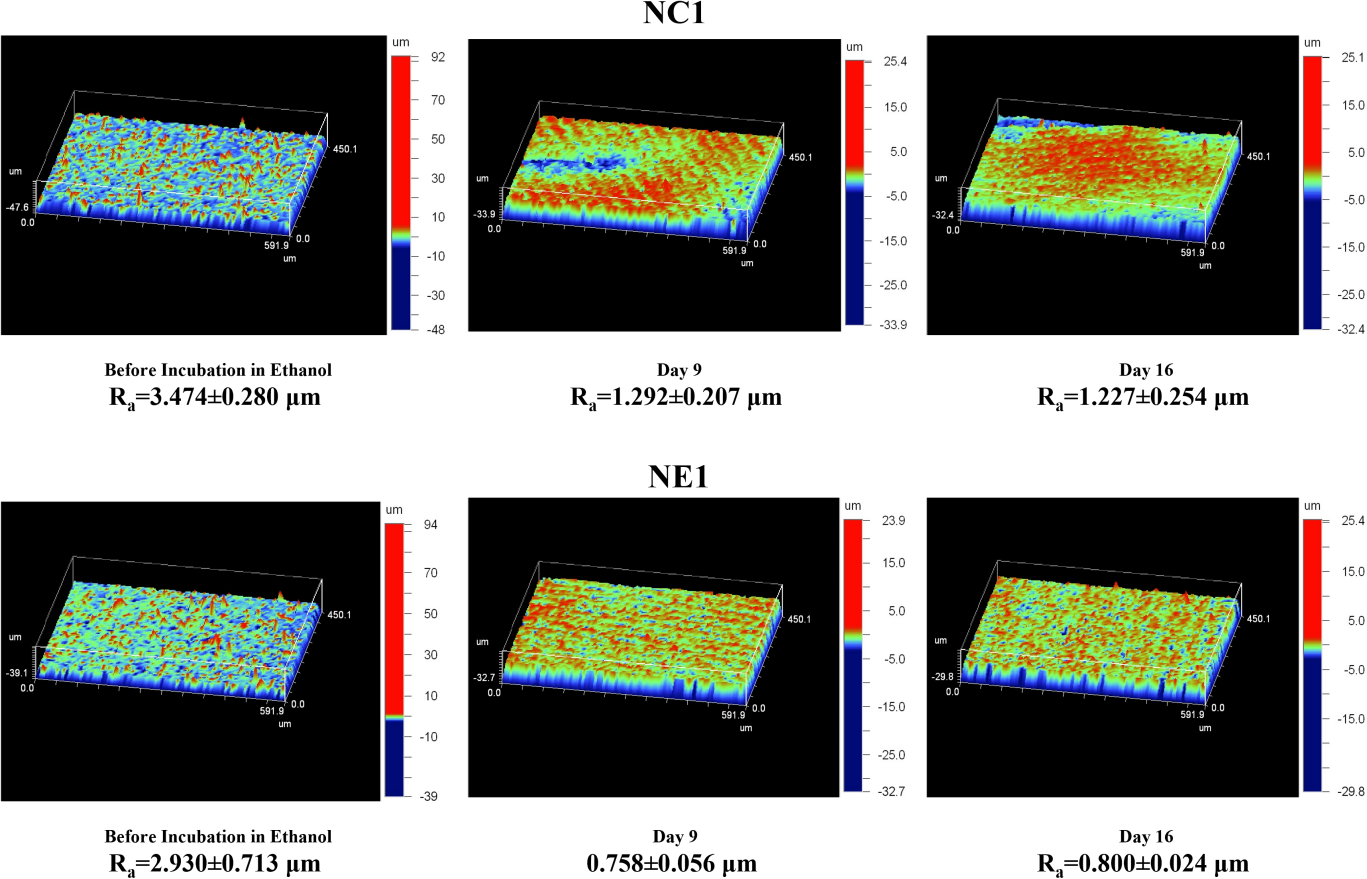
Change of surface roughness of NC1 and NE1 in ethanol incubation.

The average surface roughness (*R*_a_) of the randomly selected disc samples at baseline (before aging in ethanol) was 3.474 ± 0.280 μm and 2.930 ± 0.713 μm for NC1 and NE1, respectively (*p > 0.05*). Surface roughness for both formulations decreased following aging in ethanol. At day 9 the average surface roughness of the ethanol-stored samples was 1.292 ± 0.207 μm and 0.758 ± 0.056 μm (*p < 0.05*) for NC1 and NE1, respectively. At day 16 the average surface roughness of the ethanol-stored samples was 1.227 ± 0.254 μm and 0.800 ± 0.024 μm (*p < 0.05)* for NC1 and NE1, respectively (Figure 8; Table 2).

## 4 Discussion

Multiple strategies have been proposed to increase the resistance of adhesives to degradation. These strategies run the gamut from changing the monomer structure to exploiting the traits of biomolecules (24–28). Hydrophobicity of the monomer structure has been increased by introducing a urethane group (29–31), branched methacrylate linkage (32), or ethoxylated BisGMA (33). Other strategies include new photoinitiators and/or co-initiators (30, 34), antimicrobials (35), and enzyme-inhibitors (36–39). These diverse strategies have advanced the field and contributed significantly to our understanding of adhesive degradation and failure.

A strategy that we have found particularly promising involves self-strengthening adhesives that resist hydrolysis-mediated degradation using a mechanism that provides intrinsic reinforcement of the polymer network in both neutral and acidic conditions (14). The alkoxysilane-containing adhesives experience free radical polymerization (FRP) and sol-gel reactions. While we have reported the benefits of the self-strengthening approach (14–16), we have not used it in hydrophobic resins with high crosslink ratio and a rigid polymer backbone. These elements could work synergistically to inhibit the reactions that promote the formation of additional crosslinks and the evolution of the network structure in wet environments.

Our exploration of methacrylate-based adhesives containing alkoxysilane has revealed a complex network system that offers interesting properties and promise as a next-generation durable dental adhesive. Some of our prior research was focused on refining the photoinitiator system (14, 40, 41), other research focused on multifunction crosslinkers (41) or the synthesis of new molecules (42). In 2022, we studied hydrophilic formulations with low crosslinker concentration, i.e., 73 wt% HEMA and 15 wt% BisGMA, to analyze the effect of hydrophilicity coupled with low crosslink density on free radical polymerization and the sol-gel reaction. The mechanical properties were measured using water-saturated samples with the goal of mimicking load transfer in the wet environment of the mouth. The results indicated that the sol-gel reaction was facilitated by the ready transport of water in the hydrophilic resin (16). Our 2020 publication was focused on the time-dependent mechanical properties of methacrylate-based adhesives with autonomous strengthening capabilities (15). The results from the stress relaxation test and the dynamic mechanical analyses suggested that the network structure of the alkoxysilane-containing adhesive evolved during aging in water. Overall, the network structure exhibited enhanced deformation resistance over an extended period as the autonomous strengthening reaction propagated.

The current investigation of the alkoxysilane-containing adhesives was prompted, in part, by recent observations that hydrophobic resins offer significant benefits for vulnerable composite/tooth interfacial margins (18). We postulated that hydrophobicity coupled with high crosslink density would inhibit the sol-gel reaction and concomitantly, autonomous strengthening properties. The results of the dynamic mechanical analyses and HPLC analyses of species leached from ethanol-aged samples support the sol-gel reaction in the hydrophobic formulations. Hydrophobicity coupled with the sol-gel reaction led to enhanced mechanical properties and significant decrease in leached species, i.e., HEMA, BisGMA, and EDMAB in NE1.

As shown in Figure 1, the formation of Si-O-Si bonds is indicated for NE1. In our 2016 study, the formation of siloxane bonds (Si-O-Si) in formulations prepared with the same components but in different proportions was examined in detail (14). In the 2016 study, we reported that siloxane bonds can be detected by FTIR analysis, but these spectral features are only apparent at a suitable S/N ratio in formulations containing more than 50% silane monomers. In the current study, the properties of a hydrophobic formulation containing 15wt% silane monomer was studied and as noted previously, at this ratio the Si-O-Si bonds could not be resolved in the FTIR spectra.

### 4.1 Water sorption and contact angle

In the present study, the concentration of BisGMA was increased to determine the effect of chain mobility and hydrophobicity on the properties of adhesives with or without γ-methacryloxypropyl trimethoxy silane (MPS). The control adhesive contained methacryloxyethoxy trimethyl silane (MES).

The reduced water sorption in the current investigation as compared to our previous studies was related primarily to the increased concentration of the hydrophobic crosslinker, BisGMA. For example, in the current study the concentration of BisGMA was 55 wt% and water sorption 7.82 and 7.28% for the NC1 and NE1 adhesives, respectively. In our earlier study, the concentration of BisGMA was 15 wt% and values for water sorption were about 24% and 19% for the NC1 and NE1 adhesives, respectively. In addition, the NC1 and NE1 adhesives in our earlier study contained 10 wt% MES and MPS, respectively (15, 16).

The contact angle values highlight differences in the surface interactions of NC1 and NE1. While NC1 and NE1 contain the same HEMA/BisGMA ratios, the higher contact angle with NE1 supports additional silane-based crosslinks at the surface of this formulation. The contact angle values suggest that the silane-based crosslinks in NE1 have a significant impact on the surface.

### 4.2 Polymerization behavior

Monomer to polymer conversion is an important factor in the quality of bulk adhesives. The free radical polymerization of methacrylate-based adhesives has been thoroughly reviewed in the literature. Briefly, when the methacrylate-based adhesive is irradiated with visible light, the free radicals are generated via electron-proton transfer between the excited photosensitizer CQ and the amine EDMAB. The free radicals generated during this process enable the polymerization of methacrylate monomers (14).

In the current investigation, the band ratio profile 1,637 cm^−1^ (C = C)/1,715 cm^−1^ (C = O) was monitored to determine the conversion of the methacrylic double bond. The degree of conversion of the NC1 and NE1 is comparable at 65.4% and 65.7%, respectively. The significant decrease of 1,637 cm^−1^ methacrylate peak for both resins clearly shows the polymerization in the first three hours. These results indicate that the difference in the type of organosilanes (MES and MPS) did not affect the conversion of C = C double bonds. While the results are aligned with our earlier studies, the degree of conversion is lower for both NC1 and NE1 formulations than the formulations containing15 wt% BisGMA. The real-time degree of conversion for the NC1 and NE1 formulations containing 15 wt% BisGMA was 76% and 80%, respectively. The differences in the results between the two studies could be related to the higher viscosity and reduced chain mobility of the active molecules in the formulation containing 55 wt% BisGMA (42). As stated in our previous studies, when the silane monomer ratio in the cross-linked network decreases below 50%, detection of siloxane bonds by FTIR is obscure (14). However, the observation of a significantly increased peak at 1,075 cm^−1^ within the first three hours after photopolymerization indicates the formation of siloxane bonds despite the low rates in Figure 2. It was determined that such bond formation was not observed in the silane-free NC1 network structure.

### 4.3 Dynamic mechanical properties of the NC1 and NE1 specimens in dry and wet conditions

The tan δ values as a function of temperature and the average values of the peak maxima as glass transition temperatures (*T*_g_) of vacuum-dried and water-submerged NC1 and NE1 samples are shown in Figure 4. The limited temperature range for the water-submerged tests prevented complete peak formation, but the change in tan δ values with increasing temperature matched the profile of the dry samples (Figures 4A,B). At lower temperatures and in dry conditions, both formulations showed a shoulder (Figure 4A) which may be associated with the relaxation of chain segments for different crosslinked regions of the resins. The intensity of the tan δ curve is much higher for the control—indicating higher chain mobility for the control formulation. The NE1 shows more elastic behavior than the control, results that support the higher crosslink density of the NE1 (41).

The *T*_g_ values of the NE1 adhesives were significantly (*p* < .001) higher (159.8 ± 4.6°C) than those of the control samples (133.9 ± 3.8°C). In our previous study, similar resin compositions were used with a lower crosslinker concentration, and as expected, the *T*_g_ values increased (around 20°C) by increasing the crosslinker concentration (15, 16). Another approach to better understand the tan δ vs. temperature plots is to analyze the full width of the peak at half maximum (FWHM) intensity (Figure 5), which provides information about the heterogeneity of the crosslinked networks. When the NE1 samples were compared to the control, a significantly (*p* < .001) higher FWHM value was observed, indicating increased heterogeneity of the polymer network in the NE1 samples.

The plots of storage modulus vs. temperature (Figures 4C,D; Table 3) showed a decrease in storage modulus values with increasing temperature for both dry and wet conditions. At lower temperatures, the average storage moduli are comparable, and the control formulation demonstrated higher stiffness than the NE1 when dry. However, when submerged in wet conditions, the NC1 (362.1 MPa) softens significantly and more quickly at elevated temperatures (70°C) than the NE1 (1,007.2 MPa). This difference is related, in part, to the increased concentration of unreacted HEMA leached from NC1. The leached unreacted HEMA will act as a plasticizer to soften the polymer.

When Figure 4C was examined for vacuum-dried samples following the glass transition region above 100°C, the storage modulus for all samples decreased noticeably, reaching the rubbery region, and stabilizing for the remainder of the temperature range. In the rubbery region the 175°C storage modulus of NE1 (202.0 MPa) is significantly greater than that of NC1 (36.5 MPa). The relative crosslinking densities were compared using ζ values. The ζ values are calculated as the inverse ratio of the modulus in the rubbery region to the temperature (22, 43). As shown in Figure 5, the ζ values are significantly (*p* < .001) lower for NE1 (0.22 × 10^−5^ Pa^−1^K) than for NC1 (1.25 × 10^−5^ Pa^−1^K). Since lower ζ values indicate higher crosslinking in the polymer network, these findings support the additional crosslinking contribution of the sol-gel reaction in the NE1 system (15, 16).

With the data from TGA analysis of both resins (Figure 6), it can clearly be seen that the thermal resistance of NC1 and NE1 is higher than the application temperatures of dental adhesives. Importantly, the initiation of degradation for both resin formulations occurs at significantly higher temperatures than those applied during Dynamic Mechanical Analysis (DMA), underscoring the suitability of the temperature range chosen for DMA. This demonstrates that the DMA was performed well within the thermal safety window of the resins, ensuring that the analysis did not compromise the integrity of the resin samples due to thermal degradation.

### 4.4 Leaching properties by high-performance liquid chromatography (HPLC) studies and mechanism of network structure evolution

We used ethanol which is not a clinically relevant solvent to both accelerate the leaching and enable the release of hydrophobic degradants such as BisGMA. Using ethanol as the degradative solvent sheds light on those monomers and/or oligomers that could leach under aggressive conditions (16, 44, 45). HPLC data obtained from specimens stored in ethanol are expected to yield a high cumulative concentration of leachates as compared to clinically relevant conditions.

The highest concentration of leachate in the ethanol-stored samples is HEMA for both NC1 and NE1 (Figure 7). The concentration of HEMA leached from NC1 is four-fold greater than the amount leached from NE1. The concentration of leached EDMAB and BisGMA is greater for NC1 than NE1. The concentration of leached EDMAB is five-fold greater for NC1. The concentration of BisGMA leached from NC1 is six-fold greater than the concentration of BisGMA leached from NE1. The analyses of degradants from ethanol-aged NC1 and NE1 formulations support additional crosslinking of the polymer network in the wet environment as a result of the sol gel reaction.

The surface roughness of both formulations decreased significantly after ethanol aging (Figure 8). The decrease is related to swelling of the surface. Interestingly, the surface roughness values show a similar trend to the degradant data. There is a 37% decrease in the surface roughness of NC1 between baseline and day 9 of ethanol aging. In comparison, there is a 26% decrease in the surface roughness of NE1 over this same period. The differences in the surface roughness suggest that the NE1 formulation resists swelling as a result of increased crosslinking (Table 2).

## 5 Conclusion

The composite-restoration margin where the adhesive is applied is vulnerable to recurrent decay, fracture, and detachment—actions that will ultimately lead to composite restoration failure. The structure of methacrylate adhesives suggests a general mechanism for their degradation in the mouth (46). Water that is trapped within the adhesive or water that infiltrates porosities in the adhesive facilitates leaching of unreacted monomers (47–50). Mechanical wear of adhesive exposed at the gingival margin further accelerates degradation—wear disrupts the integrity of the adhesive surface and water will readily infiltrate the disturbed surface. Water plasticizes the polymer matrix and promotes chemical hydrolysis of ester bonds (51)—the ester bonds have been called the chemical “Achilles heel” of methacrylate adhesives (52).

We have explored adhesive formulations that will potentially thrive in the wet environment by providing intrinsic reinforcement of the polymer network. While we have reported the benefits of this self-strengthening approach (14–16), the current study is the first to investigate self-strengthening in hydrophobic resin formulations with high crosslinker ratios and a rigid polymer backbone. This combination could inhibit the reactions that promote the formation of additional crosslinks and the evolution of the network structure in wet environments. To test this hypothesis, we developed and systematically characterized hydrophobic methacrylate-based resin formulations with (NE1) and without (NC1) γ-methacryloxypropyl trimethoxy silane (MPS). The dynamic mechanical properties were studied under both dry and wet conditions at 37 and 70°C. The results of the mechanical testing provide evidence of intrinsic reinforcement and increased crosslinking in wet environments for the NE1 formulation. Analyses of the degradants from ethanol-aged samples support increased crosslinking density in the NE1 polymer in the wet environment. The increased crosslinking density of the NE1 leads to a four- and sixfold reduction in leached HEMA and BisGMA.

In summary, the results indicate that free radical polymerization and sol gel reaction coupled with hydrophobicity in NE1 leads to a significant decrease in leachates under aggressive aging conditions and reduced deterioration of mechanical properties at elevated temperatures under wet conditions. This behavior is achieved without inhibiting monomer-to-polymer conversion or reducing polymerization kinetics. While the results are promising, there are limitations, e.g., the hydrophobic resin may not infiltrate the wet, demineralized dentin matrix. This potential limitation requires further investigation.

In conclusion, high cross-link density and self-strengthening polymers show great promise as a new generation dentin adhesive. In this context, increased crosslinking density and the self-reinforcing ability of the polymer can potentially increase the durability and performance of dentin adhesives in the wet environment of the mouth.

## Data Availability

The raw data supporting the conclusions of this article will be made available by the authors, without undue reservation.
